# Homotherapy for heteropathy: therapeutic effect of Butein in NLRP3-driven diseases

**DOI:** 10.1186/s12964-024-01695-7

**Published:** 2024-06-07

**Authors:** Wenhao Liao, Yuchen Li, Jingwen Liu, Yu Mou, Mei Zhao, Juan Liu, Tianxin Zhang, Qin Sun, Jianyuan Tang, Zhilei Wang

**Affiliations:** 1 TCM Regulating Metabolic Diseases Key Laboratory of Sichuan Province, Hospital of Chengdu University of Traditional Chinese Medicine, Chengdu, 610075 China; 2 Hospital of Chengdu University of Traditional Chinese Medicine, Chengdu, 610075 China; 3 School of Basic Medical Sciences, Chengdu University of Traditional Chinese Medicine, Chengdu, 610075 China; 4 National Traditional Chinese Medicine Clinical Research Base of the Affiliated Traditional Chinese Medicine, Hospital of Southwest Medical University, Luzhou, 646000 China

**Keywords:** Butein, NLRP3 inflammasome, Nrf2, Colitis, Non-alcoholic steatohepatitis, Peritonitis

## Abstract

**Background:**

Aberrant inflammatory responses drive the initiation and progression of various diseases, and hyperactivation of NLRP3 inflammasome is a key pathogenetic mechanism. Pharmacological inhibitors of NLRP3 represent a potential therapy for treating these diseases but are not yet clinically available. The natural product butein has excellent anti-inflammatory activity, but its potential mechanisms remain to be investigated. In this study, we aimed to evaluate the ability of butein to block NLRP3 inflammasome activation and the ameliorative effects of butein on NLRP3-driven diseases.

**Methods:**

Lipopolysaccharide (LPS)-primed bone-marrow-derived macrophages were pretreated with butein and various inflammasome stimuli. Intracellular potassium levels, ASC oligomerization and reactive oxygen species production were also detected to evaluate the regulatory mechanisms of butein. Moreover, mouse models of LPS-induced peritonitis, dextran sodium sulfate-induced colitis, and high-fat diet-induced non-alcoholic steatohepatitis were used to test whether butein has protective effects on these NLRP3-driven diseases.

**Results:**

Butein blocks NLRP3 inflammasome activation in mouse macrophages by inhibiting ASC oligomerization, suppressing reactive oxygen species production, and upregulating the expression of the antioxidant pathway nuclear factor erythroid 2-related factor 2 (Nrf2). Importantly, in vivo experiments demonstrated that butein administration has a significant protective effect on the mouse models of LPS-induced peritonitis, dextran sodium sulfate-induced colitis, and high-fat diet-induced non-alcoholic steatohepatitis.

**Conclusion:**

Our study illustrates the connotation of homotherapy for heteropathy, i.e., the application of butein to broaden therapeutic approaches and treat multiple inflammatory diseases driven by NLRP3.

**Supplementary Information:**

The online version contains supplementary material available at 10.1186/s12964-024-01695-7.

## Graphical Abstract

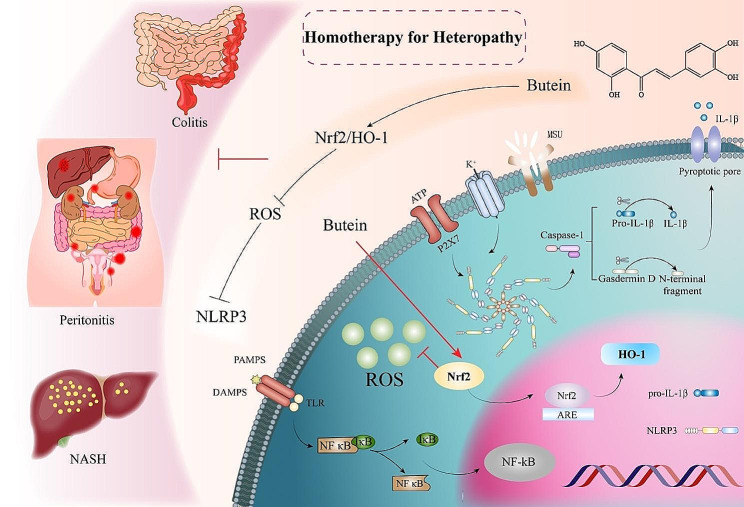

## Background

Homotherapy for heteropathy refers to the principle that different diseases, due to the same pathogenesis in their development process, adopt the same treatment method [1, 2]. Metformin, for example, possesses a combination of hypoglycemic, weight loss, anti-tumor, anti-aging, and cardiovascular effects, with the underlying common mechanism being that metformin acts directly on its molecular target, presenilin enhancer 2 (PEN2), which in turn targets the lysosomal AMP-activated protein kinase pathway to achieve metabolic regulation [3]. Similarly, inflammation plays a key role in diseases such as infection, inflammatory bowel disease, non-alcoholic steatohepatitis (NASH), Alzheimer’s disease, atherosclerosis, and type 2 diabetes, where hyperactivation of inflammasome is one of the key pathogenetic mechanisms, suggesting that targeting inflammasome may be a common approach to the treatment of these complex diseases [4–7].

Inflammasomes, multiprotein complexes with pro-inflammatory effects, are essential components of the innate immune response by recognizing different molecules, known as pathogen- or damage-associated molecular patterns, or disrupting cellular homeostasis [8]. The NOD-like receptor family, nucleotide-binding, oligomerization domain-like receptor family pyrin domain containing 3 (NLRP3) forms an inflammasome complex with apoptosis-associated speck-like protein containing a CARD (ASC) and caspase 1 in response to a variety of endogenous and exogenous stimuli, such as pathogens, dead cells, or environmental stimuli [6]. Once the NLRP3 inflammasome is assembled, caspase 1 self-cleaves to form active caspase 1, which in turn cleaves pro-IL-1β and pro-IL-18 into active IL-1β and IL-18, respectively [9–11]. Furthermore, the active form of caspase 1 cleaves the protein gasdermin D (GSDMD) into C-GSDMD and N-GSDMD. N-GSDMD migrates and anchors to the cell membrane, where it forms a pore that initiates pyroptosis, a programmed cell death, resulting in excessive inflammatory responses and host damage [12]. NLRP3 inflammasome has been implicated in the pathogenesis of a wide range of diseases, including chronic inflammatory diseases, cardiovascular diseases, autoimmune diseases, and neurodegenerative diseases associated with lifestyle and dietary changes, aging, and environmental exposures [13]. Given the potent proinflammatory potential of NLRP3 and its involvement in these serious diseases, current pharmacological targeting of NLRP3 has underscored the potential and importance of NLRP3 as a drug target [14]. However, there is no clinical application of NLRP3 inhibitors at present due to potential toxicity and other reasons. The discovery of new potential therapeutic inhibitors of NLRP3 that are safe, effective and have fewer side effects will aid in the development of common prevention and treatment strategies for various diseases.

Butein (2’,3,4,4’ – tetrahydroxychalcone) is a bioactive flavone found naturally in plants such as *Toxicodendron vernicifluum* and *Rhus verniciflua* [15–18]. Butein has excellent anti-inflammatory, antioxidant, anticancer, and anti-angiogenesis activities [19, 20]. Butein reduces the production of various pro-inflammatory mediators, such as TNF-α, PGE-2, COX, and PGE-2, by modulating the NF-κB [21], PI3K/Akt [17], and JNK [22] signaling pathways. In addition, butein inhibits ROS production and increases antioxidant gene expression by activating nuclear factor erythroid 2-related factor 2 (Nrf2) to attenuate oxidative stress and inflammation [15–18]. Although butein exhibits significant beneficial effects, its potential mechanisms and direct targets remain to be investigated. In this study, we speculate that butein may achieve homotherapy for heteropathy in peritonitis, ulcerative colitis, and NASH by suppressing NLRP3 inflammasome activation. We focused on the ability of butein to block NLRP3 inflammasome activation in mouse macrophages and evaluated the ameliorative effects of butein on lipopolysaccharide (LPS)-induced peritonitis, dextran sodium sulfate (DSS)-induced colitis, and high-fat diet (HFD)-induced NASH models. These findings are expected to deepen the connotation of heterogeneous diseases and expand the potential candidates of butein as a treatment for NLRP3-driven diseases and its promising use in clinical practice.

## Materials and methods

### Mice

Male C57BL/6 mice (6–8 weeks old) were purchased from Chengdu Yaokang Biotechnology Co., Ltd. (Chengdu, China). All mice were specific pathogen-free, maintained under 12 h of light/dark conditions at 22–24 °C and 40–60% relative humidity, with unrestricted access to food and water for the duration of the experiment. All animal experiment protocols in this study were conducted in accordance with the Guidelines for the Care and Use of Laboratory Animals and approved by the Animal Research Ethics Society of Chengdu University of Traditional Chinese Medicine (No. 2,024,024).

### Cell culture

Mouse bone marrow-derived macrophages (BMDMs) were prepared as follows. The femurs of adult C57BL/6 mice were removed, and the bone marrow contents were repeatedly rinsed with Dulbecco’s modified Eagle’s medium (DMEM, Gibco, 11,965,092) supplemented with 10% fetal bovine serum (Gibco, 10,100), and 1% penicillin/streptomycin (Solarbio, P1400). At the same time, 50 ng/mL recombinant murine macrophage colony-stimulating factor (MedChemExpress, HY-P7085) was added to the medium, and the cells were placed in a 5% CO_2_ atmosphere in a humidified incubator. On Day 5 of cell culture, BMDMs were resuspended and inoculated into culture plates at a density of 1 × 10^6^ cells/mL for subsequent experiments.

### Inflammasomes activation

The methods used to induce inflammasomes activation have been described previously [23]. Briefly, BMDMs were seeded at a density of 1 × 10^6^ cells/mL in 12-well plates overnight, followed by stimulation with 50 ng/mL ultrapure LPS (Invivogen, tlrl-3pelps) for 4 h. Butein (MedChemExpress, HY-16,558, purity 99.95%) was dissolved in dimethyl sulfoxide (DMSO). The medium was then changed to serum-free Opti-MEM (Gibco, 31,985,070) containing butein (2.5, 5, or 10 µM) for 1 h, followed by treatment with ATP (5 mM, Sigma-Aldrich, A2383), or nigericin (7.5 µM, Invivogen, tlrl-nig) for 1 h; or transfection with poly(I: C) (2 µg/mL, Invivogen, tlrl-picw), or monosodium urate (MSU) crystals (250 µg/mL, Invivogen, tlrl-msu) for 6 h. Poly(dA: dT) (2 µg/mL, Invivogen, tlrl-patn) and ultrapure flagellin (100 ng/mL, Invivogen, tlrl-epstfla) were transfected with Lipo8000™ (Beyotime, C0533) to induce AIM2 and NLRC4 inflammasomes activation, respectively. To induce non-canonical NLRP3 inflammasome activation, BMDMs were primed with Pam3CSK4 (1 µg/mL, Invivogen, tlrl-pms) for 4 h, and then transfected with ultrapure LPS (1 µg/mL) with Lipo8000™ for 6 h. To investigate fatty acid [(sodium palmitate/sodium oleate (PO), Sigma, P9767/O7501)]-induced NLRP3 inflammasome activation, BMDMs were primed with LPS, followed by stimulation with PO (300 µM) for 6 h.

### Cell viability assay

BMDMs (1 × 10^5^ cells/well) were seeded in 96-well plates overnight and then exposed to butein (10, 20, 30, 40, 50, 60, 70, 80, or 90 µM) for 24 h. Cell Counting Kit-8 (CCK-8) reagent (MedChemExpress, HY-K0301) was added to the cell culture medium and incubated with the cells for 30 min. Optical density values at a wavelength of 450 nm were analyzed using a multifunctional microplate reader (Tecan, Infinite 200Pro).

### Antibodies

Anti-mouse caspase-1 (AG-20B-0042) and anti-NLRP3 (AG-20B-0014) were purchased from Adipogen. An anti-mouse IL-1β antibody (AF-401-NA) was purchased from R&D System. An anti-ASC (SC-22,514-R) was purchased from Santa Cruz Biotechnology. Anti-GSDMD (ab209845) was purchased from Abcam. Lamin B1 polyclonal antibody (12987-1-AP), NRF2 polyclonal antibody (16396-1-AP), heme oxygenase-1 (HO-1/HMOX1) polyclonal antibody (10701-1-AP), goat anti-rabbit IgG (SA00001-2), and goat anti-mouse IgG (SA00001-1) were purchased from Proteintech.

### Lactate dehydrogenase (LDH) assay

LDH secretion in culture supernatants was determined using the LDH Cytotoxicity Assay Kit (Beyotime, C0016) according to the manufacturer’s instructions. Briefly, 60 µL of each supernatant sample was collected in a 96-well plate. Then, 60 µL of LDH Cytotoxicity Assay Kit reagent was added to each well and incubated for 30 min. The absorbance signal was measured at 490 nm using a microplate reader (Tecan, Infinite 200Pro).

### Caspase-1 activity assay

Caspase-1 activity in the cell culture supernatant was assessed using the Caspase-1-Glo® 1 Inflammasome Assay according to the manufacturer’s instructions (Promega, G9951). Caspase-Glo® 1 buffer was transferred to a bottle containing the Z-WEHD substrate to reconstitute the substrate. MG-132 inhibitor was added to the reconstituted Z-WEHD substrate to prepare the Caspase-Glo® 1 reagent. An equal volume of Caspase-Glo® 1 Reagent and cell culture supernatants were then used to measure luminescence (Tecan, Infinite 200Pro).

### Enzyme-linked immunosorbent assay (ELISA)

Determination of IL-1β (Elabscience, E-MSEL-M0003), mouse TNF-α (Elabscience, E-EL-M3063), and mouse IL-6 (Dakewe, 1,210,602) in cell culture supernatants and mouse serum was performed according to the manufacturer’s instructions.

### Hoechst 33,342/propidium iodide (PI) fluorescence staining

BMDMs were stained with 5 µg/mL Hoechst 33,342 (Beyotime, C1025) for 10 min at 37 °C in the dark, followed by incubation with 10 µg/mL propidium iodide (PI, Beyotime, ST511) for 15 min at 25 °C in the dark. The cells were observed by fluorescence microscopy (Olympus, U-RFL-T).

### ASC oligomerization

After activation of inflammasome as described above, the cells were lysed with Triton buffer [50 mM Tris-HCl (pH 7.5), 150 mM NaCl, 0.5% Triton X-100, and protease inhibitor cocktail] for 30 min. The lysates were subsequently centrifuged at 6000 g for 15 min at 4 °C. The pellets were washed in 500 µL of ice-cold PBS and resuspended in 200 µL of PBS. A 2 mM disuccinimidyl suberate crosslinker (MedChemExpress, HY-W019543) was added to the resuspended pellets and incubated at 30 °C for 30 min. The samples were then centrifuged at 6000 g for 15 min at 4 °C. The cross-linked pellets were resuspended in 30 µL of 1×SDS loading buffer and then boiled for 15 min and analyzed by immunoblotting with an anti-ASC antibody.

### Intracellular potassium (K^+^) assay

BMDMs were seeded in 12-well plates overnight and stimulated as described above. The cells were then washed 3 times with saline, followed by the addition of HNO_3_ to lyse the BMDMs, and the samples were collected and boiled at 100 °C for 30 min. Intracellular K^+^ was detected by inductively coupled plasma-mass spectrometry (ICP-MS).

### Reactive oxygen species (ROS) assay

BMDMs were seeded overnight in 12-well plates and then stimulated as described above. Cells were washed twice with Hank’s balanced salt solution (HBSS) and then stained with 10 µM DCFH-DA (MedChemExpress, HY-D0940) for 20 min at 37 °C, immediately followed by two washes with HBSS. Cells were resuspended in HBSS and ROS were detected using a Navios flow cytometer (Beckman Coulter, Brea, USA).

### LPS-induced peritonitis mouse model

Butein was dissolved in saline containing 10% DMSO, 40% PEG 400 and 5% Tween-80. C57BL/6 male mice were injected intraperitoneally with butein (*n* = 10, 10 or 20 mg/kg body weight) for 1 h followed by 20 mg/kg body weight LPS [from *Escherichia coli* (O55:B5), Sigma-Aldrich, L2880] [24]. Mice were sacrificed 2 h after LPS injection, and serum was collected. The peritoneal cavity was washed with ice-cold 1× PBS, and peritoneal lavage fluid was collected. ELISAs were performed to measure the levels of IL-1β and TNF-α production in serum and peritoneal lavage fluid. A Navios flow cytometer (Beckman Coulter, Brea, USA) was used to analyze polymorphonuclear neutrophils (Ly6G^+^CD11b^+^, BioLegend, 127,613, 101,205) in the peritoneal lavage fluid.

### DSS-induced colitis mouse model

DSS (2.5% (wt/vol), MP Biomedicals, 9011-18-1) was dissolved in daily drinking water for 9 days to induce colitis [25]. Mice received butein (*n* = 10, 10 or 20 mg/kg body weight) by intragastric gavage during the modeling period. The weight, fecal consistency, and presence of blood in the feces of the mice were recorded daily. The disease activity index (DAI) score, which is used to assess and quantify the severity of intestinal damage during DSS administration, was calculated based on the composite score of diarrhea (0 points = normal, 1–2 points = soft stools, and 3–4 points = watery diarrhea), bloody stools (0 points = normal stools, 1–2 points = slight bleeding, and 3–4 points = excessive bleeding), and body weight loss (0 points = no weight loss, 1 point = 1-5% weight loss, 2 points = 6-10% weight loss, 3 points = 11-20% weight loss, and 4 points = > 20% weight loss). On Day 9 after colitis induction, the mice were necropsied, and the serum was collected to determine the IL-1β levels. The colon was removed, washed with saline, and measured for length. A portion of the colon tissue was placed in neutral formalin, and hematoxylin-eosin (HE) staining was performed to assess pathological injury. The expression of IL-1β and caspase-1 in colon tissues was analyzed by immunohistochemical staining, while the expression of inflammasome-associated proteins in colon tissues was detected by immunoblotting.

### HFD-induced NASH mouse model

After 1 week of acclimatization, 10 male C57BL/6 mice fed a regular diet (RD, XTCON50J, containing 4.3% fat, 19.2% protein, and 67.3% carbohydrate) were randomly selected as the control group. Twenty mice fed a HFD (XTHF60, containing 10% fat, 20% protein, and 70% carbohydrate) were randomized into the HFD group (*n* = 10), butein low-dose group (*n* = 5), or butein high-dose group (*n* = 5). Eight weeks later, butein (10, or 20 mg/kg body weight) was administered intragastrically for 4 weeks. Control and model mice received saline containing 10% DMSO, 40% PEG 400 and 5% Tween-80 once daily continuously for 4 weeks. Mouse body weights were recorded once a week throughout the experiment. After 12 weeks of dietary intervention, all the mice were fasted overnight.

Blood samples were collected after anesthesia, and serum IL-1β secretion was measured by ELISA. Serum triglyceride (TG, A110-1-1), total cholesterol (TC, A111-1-1), alanine aminotransferase (ALT, C009-2-1), and aspartate aminotransferase (AST, C010-2-1) levels were measured using assay kits from Nanjing Jiancheng Institute of Bioengineering according to the manufacturer’s instructions. Livers were harvested and weighed. Protein samples were extracted from liver tissues for immunoblot analysis, and liver histopathological changes were evaluated by HE staining, oil red O staining, and Masson’s staining.

### Statistical analyses

Statistical analysis was performed using Prism 8 software (GraphPad Software). All experimental data are expressed as the mean ± standard error of the mean (SEM). Significant differences were statistically evaluated using unpaired Student’s t test for two groups or one-way ANOVA followed by Dunnett’s post hoc test for multiple groups. Difference was considered statistically significant when *P* < 0.05.

## Results

### Butein inhibits caspase-1 activation and IL-1β secretion

**Fig. 1:**
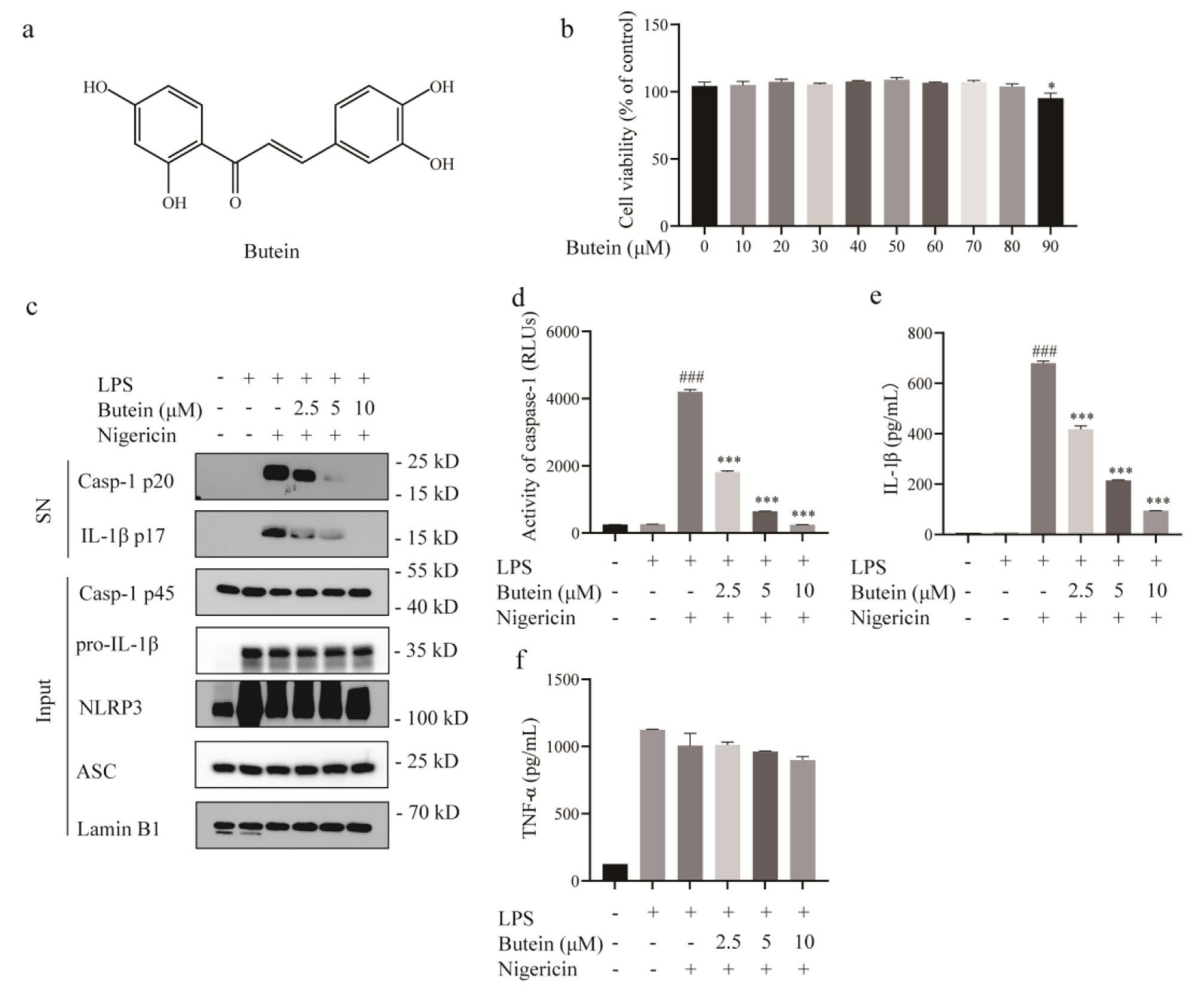
Butein inhibits caspase-1 activation and IL-1β secretion. **a** The structure of butein. **b** Cell viability of butein. **c** BMDMs were primed with LPS for 4 h and then treated with butein for 1 h before stimulation with nigericin for 1 h. Western blot analyses of pro-IL-1β, caspase-1 p45, NLRP3, and ASC in lysates (Input) and IL-1β p17, caspase-1 p20 in culture supernatants (SN). **d-f** Caspase-1 activity (**d**), IL-1β (**e**) and TNF-α (**f**) secretion were measured in the SN. ^###^*P* < 0.001 vs. the group of LPS. ^***^P < 0.001 vs. the group of LPS + nigericin

To assess whether butein (Fig. 1a) affects the activation of NLRP3 inflammasome, we first tested the cytotoxicity of butein. When different doses of butein were applied to BMDMs for 24 h, it was observed that 0–80 µM butein had no apparent cytotoxic effect (Fig. 1b). Therefore, we next measured the effect of 0–10 µM butein on inflammasome activation. BMDMs were first primed with LPS and then treated with butein (2.5, 5, or 10 µM) for 1 h before stimulation with nigericin. Compared with the untreated BMDMs, LPS-primed BMDMs expressed high levels of NLRP3 and pro-IL-1β. After stimulation with nigericin, butein markedly blocked the cleavage of caspase-1 and IL-1β to their mature p20 and p17 forms, respectively (Fig. 1c and Supplementary Fig. 1a). Furthermore, the increase in caspase-1 activity triggered by nigericin in cell supernatants was enhanced but was effectively inhibited by butein in a dose-dependent manner (Fig. 1d). The ELISA results also showed that butein decreased IL-1β secretion in a concentration-dependent manner (Fig. 1e). However, the production of TNF-α, an inflammasome-independent cytokine, was not affected by butein (Fig. 1f). In conclusion, butein effectively blocked nigericin-induced NLRP3 inflammasome activation.

### Butein prevents caspase-1-dependent GSDMD cleavage and pyroptosis

**Fig. 2:**
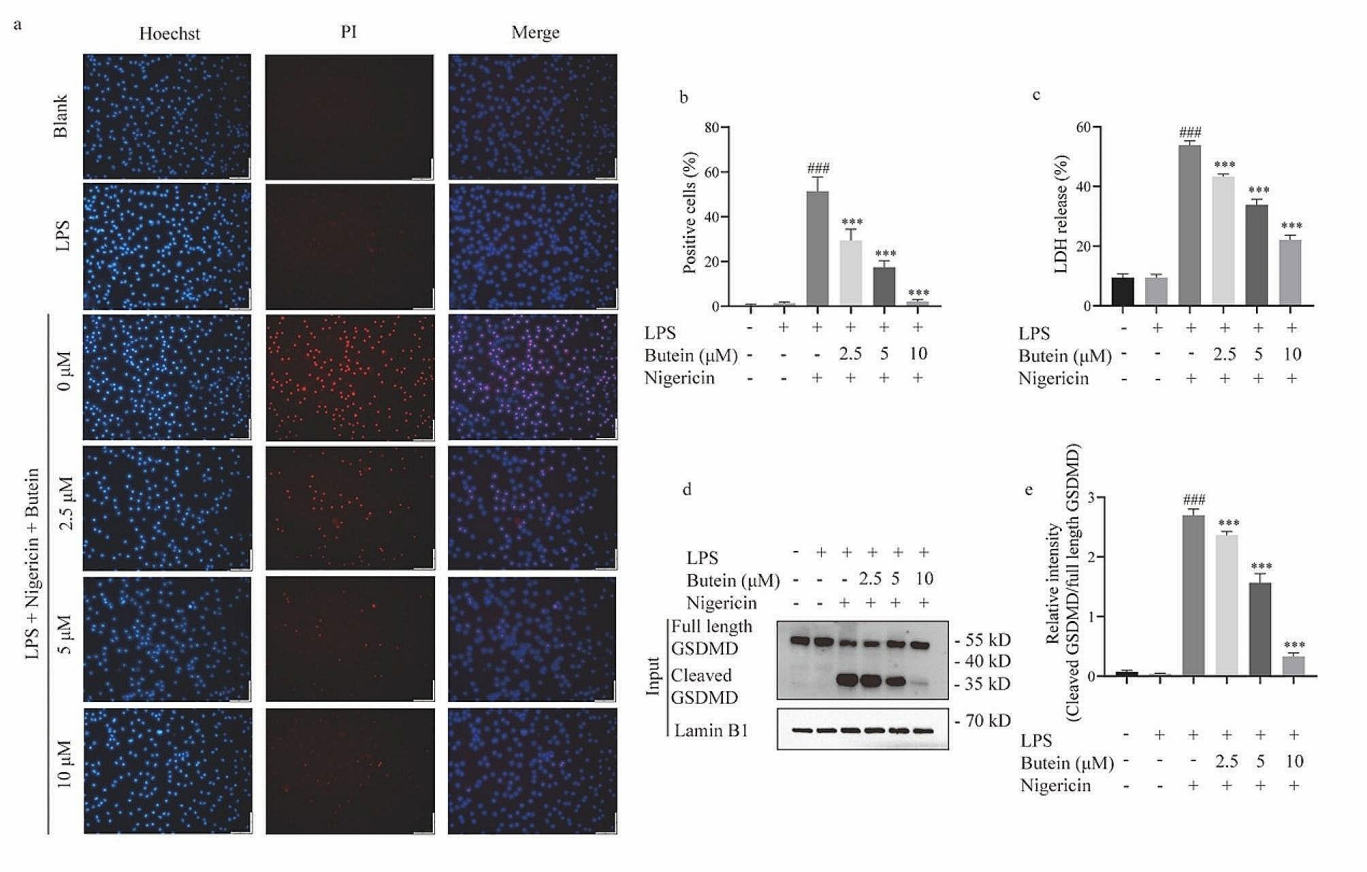
Butein prevents caspase-1-dependent GSDMD cleavage and pyroptosis. **a, b** LPS-primed BMDMs were treated with butein for 1 h prior to stimulation with nigericin for 1 h. BMDMs were then stained with Hoechst 33,342 followed by PI. Cells were observed by fluorescence microscope (**a**), and the PI-positive cells were counted (**b**). **c** Effect of butein on LDH release. **d** Western blot analysis of cleaved GSDMD in the input of BMDMs. **e** Relative intensity of cleaved GSDMD described in (**d**). ^###^*P* < 0.001 vs. the group of LPS. ^***^P < 0.001 vs. the group of LPS + nigericin

Pyroptosis is a type of programmed cell death that depends on caspases (mainly caspase-1, 4, 5, and 11) and activates a strong inflammatory response [26]. We further investigated the regulatory role of butein in pyroptosis. The results showed that incubation of BMDMs with LPS and nigericin resulted in pyroptosis, characterized by a significant increase in PI staining (Fig. 2a, b). Butein dramatically reduced the number of PI-positive cells in a dose-dependent manner (Fig. 2a, b). In addition, LDH release in the supernatant was measured as a parameter of pyroptosis. The results showed that butein observably blocked nigericin-induced LDH release (Fig. 2c). GSDMD was identified as a cleavage target of caspase-1 and is required for pyroptosis and IL-1β secretion. In nigericin-stimulated BMDMs, the level of cleaved GSDMD decreased after butein treatment in a concentration-dependent manner (Fig. 2d, e). Taken together, these results suggest that butein is an excellent inhibitor of pyroptosis through the prevention of NLRP3 inflammasome-mediated caspase-1 activation.

### Butein blocks multiple agonist-induced NLRP3 inflammasome activation

**Fig. 3:**
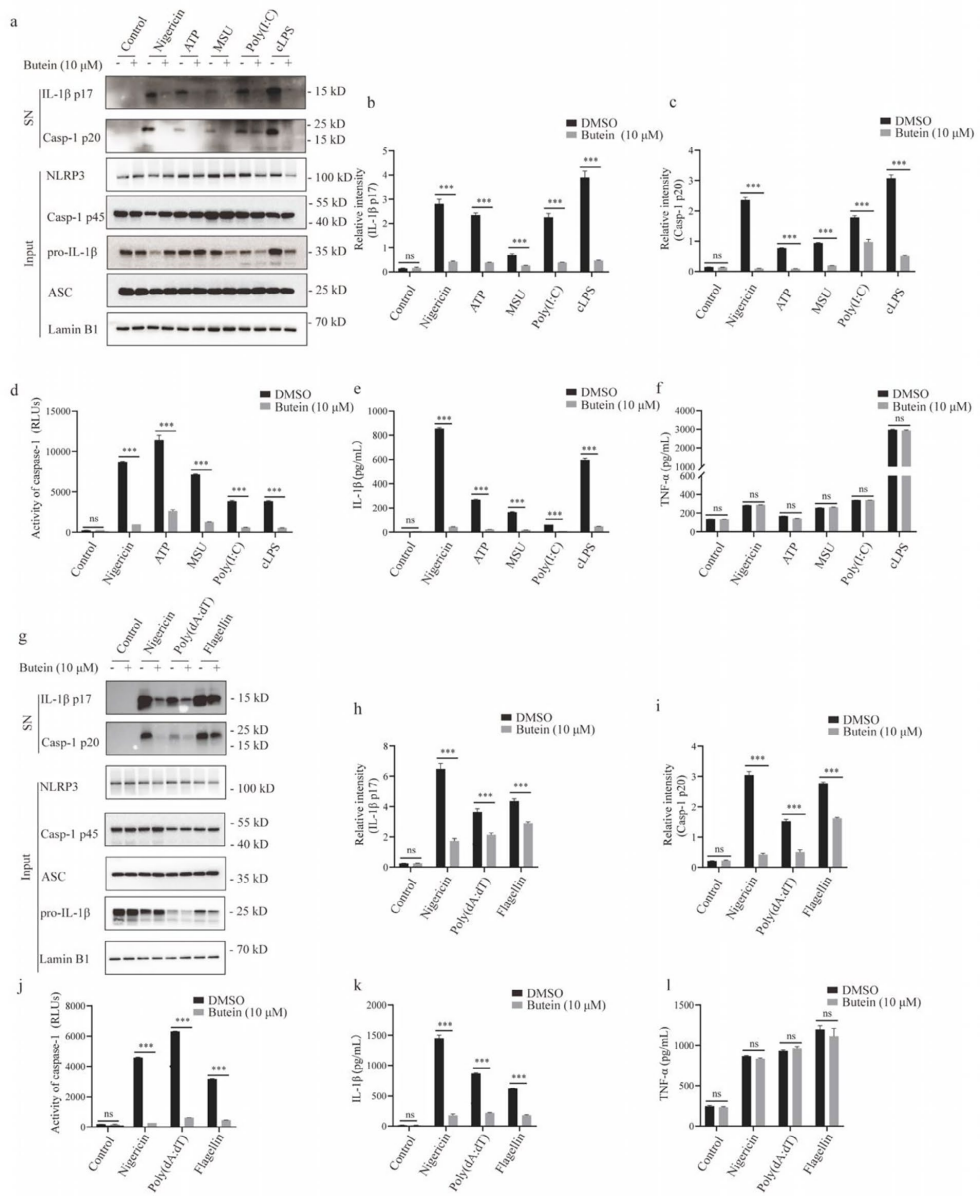
Butein blocks multiple agonist-induced inflammasomes activation. **a** LPS-primed BMDMs were treated with butein prior to stimulation with nigericin, ATP, MSU, poly(I: C), or Pam3CSK4-primed BMDMs were treated with butein and followed by transfection with ultrapure LPS. Western blot analyses of pro-IL-1β, caspase-1 p45, NLRP3, and ASC in input and IL-1β p17, caspase-1 p20 in SN. **b, c** Relative intensity of IL-1β p17 (**b**) and caspase-1 p20 (**c**) described in (**a**). **d-f** Caspase-1 activity (**d**), IL-1β (**e**) and TNF-α (**f**) secretion were measured in the SN. **g** LPS-primed BMDMs were treated with butein before stimulation with nigericin, poly (dA: dT), and flagellin. Western blot analyses of pro-IL-1β, caspase-1 p45, NLRP3, and ASC in input and IL-1β p17, caspase-1 p20 in SN of BMDMs. **h, i** Relative intensity of IL-1β p17 (**h**) and caspase-1 p20 (**i**) described in (**g**). **j-l** Caspase-1 activity (**j**), IL-1β (**k**) and TNF-α (**l**) secretion were measured in the SN. ^***^P < 0.001

Next, we tested the effect of butein on the activation of NLRP3 inflammasome induced by other stimuli. LPS-primed BMDMs were treated with butein and subsequently stimulated with ATP, MSU, or poly(I: C). Similar to nigericin, ATP, MSU, and poly(I: C) induced IL-1β production and caspase-1 cleavage, whereas butein blocked the canonical activation of NLRP3 inflammasome induced by these stimuli (Fig. 3a-e). Intracellular LPS can activate caspase-11, which initiates non-canonical NLRP3 inflammasome activation. We then investigated the effect of butein on the non-canonical NLRP3 pathway. The results demonstrated that ultrapure LPS transfection induced caspase-11-dependent caspase-1 cleavage and IL-1β secretion in Pam3CSK4-primed BMDMs, whereas pretreatment with butein before intracellular LPS stimulation blocked non-canonical NLRP3 inflammasome activation (Fig. 3a-e). However, the production of TNF-α was not affected by butein (Fig. 3f). These data suggest that butein is a potent and broad inhibitor of both the canonical and non-canonical NLRP3 inflammasome.

### Butein suppresses the activation of AIM2 and NLRC4 inflammasomes

AIM2 and NLRC4 inflammasomes also mediate the secretion of IL-1β and the maturation of caspase-1, which are activated by double-stranded DNA poly(dA: dT) and flagellin, respectively, and play key roles in the progression of inflammatory diseases [27]. To elucidate whether the inhibitory effect of butein is specific to the NLRP3 inflammasome or more general, we investigated the effect of butein on the AIM2 and NLRC4 inflammasomes. The results showed that butein also inhibited IL-1β secretion and caspase-1 maturation during AIM2 and NLRC4 inflammasome activation in BMDMs (Fig. 3g-k). Meanwhile, butein did not affect TNF-α production (Fig. 3l). These results suggest that butein has a broad range of inhibitory effects on inflammasomes, including NLRP3, NLRC4, and AIM2.

### Butein inhibits NLRP3 inflammasome assembly

**Fig. 4:**
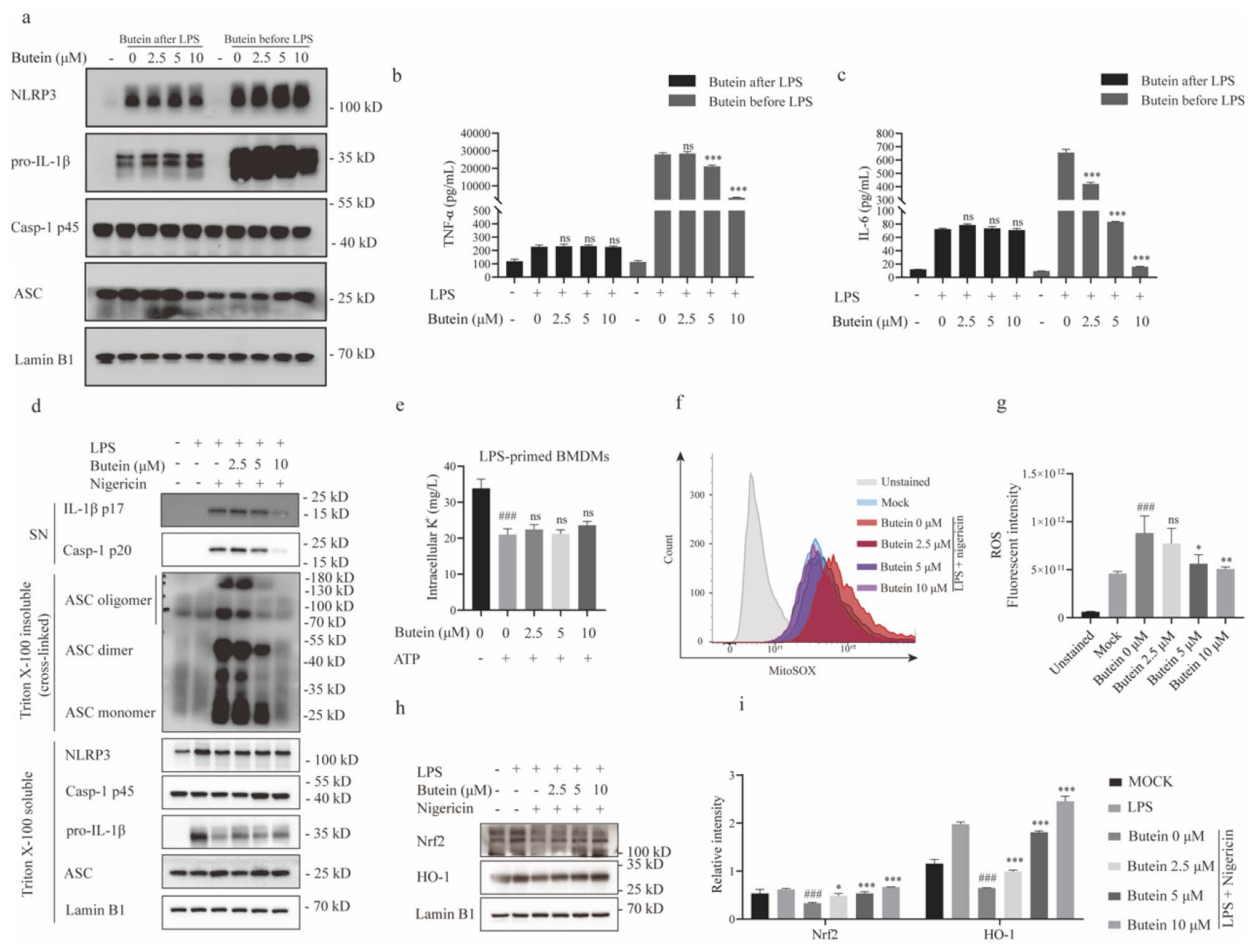
Butein inhibits NLRP3 inflammasome assembly. **a** BMDMs were treated with LPS for 4 h and then stimulated with butein for 1 h (butein after LPS) or treated with butein for 1 h and then stimulated with LPS for 4 h (butein before LPS). Western blot analysis of pro-IL-1β, caspase-1 p45, NLRP3, ASC in input. **b, c** TNF-α (**b**) and IL-6 (**c**) secretion in SN was measured by ELISA. **d** LPS-primed BMDMs were treated with butein for 1 h and then stimulated with nigericin for 1 h. Western blot analysis of cross-linked ASC in the Triton X-insoluble pellet. **e** LPS-primed BMDMs were treated with butein and then stimulated with nigericin, followed by the addition of HNO_3_ to lyse the BMDMs. Intracellular K^+^ was detected by ICP-MS. **f, g** Detection of ROS by flow cytometry (**f**), and the intracellular ROS were counted (**g**). **h, i** Western blot analyses of Nrf2 and HO-1 levels in BMDMs (**h**), and the relative intensity of Nrf2 and HO-1 (**i**). ^###^*P* < 0.001 vs. the group of LPS. ^*^P < 0.05, and ^***^P < 0.001 vs. the group of LPS + nigericin

We then investigated the mechanism by which butein inhibits NLRP3 inflammasome activation. Canonical activation of the NLRP3 inflammasome is a two-step process consisting of priming and an activation steps. Previous studies have shown that butein can inhibit NF-κB activation [28, 29], and we then examined whether butein affects LPS-induced priming for inflammasome activation. The results showed that administration of butein before LPS treatment had a slight inhibitory effect on the expression of pro-IL-1β and the production of TNF-α and IL-6. However, butein administration after LPS treatment did not affect the pro-IL-1β expression or TNF-α or IL-6 release (Fig. 4a-c), suggesting that butein does not effectively inhibit LPS-induced priming at these doses.

Since ASC oligomerization is a critical step in the recruitment and subsequent activation of caspase-1, we investigated whether butein inhibits NLRP3 inflammasome activation by inhibiting ASC oligomerization. We stimulated LPS-pretreated BMDMs with nigericin and detected ASC monomers and higher complexes by Western blotting. The results showed that butein dose-dependently inhibited nigericin-stimulated ASC oligomerization (Fig. 4d and Supplementary Fig. 1b). These results suggest that butein may have a direct effect on the oligomerization of ASC or on its upstream events during NLRP3 inflammasome assembly.

### Butein reduces ROS generation and upregulates Nrf2 signaling

Potassium (K^+^) efflux is recognized as an important trigger for the induction of NLRP3 inflammasome activation [30, 31]. Therefore, we tested whether butein prevents K^+^ efflux during NLRP3 activation. A strong decrease in intracellular K^+^ was observed when LPS-stimulated BMDMs were treated with nigericin, but this decrease was also observed when the BMDMs were pretreated with butein (Fig. 4e). This finding suggests that K^+^ efflux is not an upstream mechanism by which butein inhibits NLRP3 inflammasome activation. There is substantial evidence that oxidative stress-induced mitochondrial dysfunction and ROS generation are also involved in the upstream processes of NLRP3 inflammasome activation [14]. We found that nigericin treatment stimulated ROS generation, whereas butein reduced ROS levels in a concentration-dependent manner (Fig. 4f, g). The Nrf2/HO-1 pathway is a key regulator of cellular antioxidant responses, and Nrf2 is an intranuclear antioxidant element that binds to downstream HO-1 proteins that enter the nucleus to stimulate oxidative stress pathways [32]. Therefore, activation of Nrf2 is essential for inhibiting ROS production and managing oxidative damage. Our results showed that butein treatment reversed the decrease in the expression of Nrf2 and HO-1 (Fig. 4h, i) after nigericin stimulation, suggesting that the antioxidant effect of butein depends on the Nrf2 pathway.

### Butein alleviates DSS-induced colitis

**Fig. 5:**
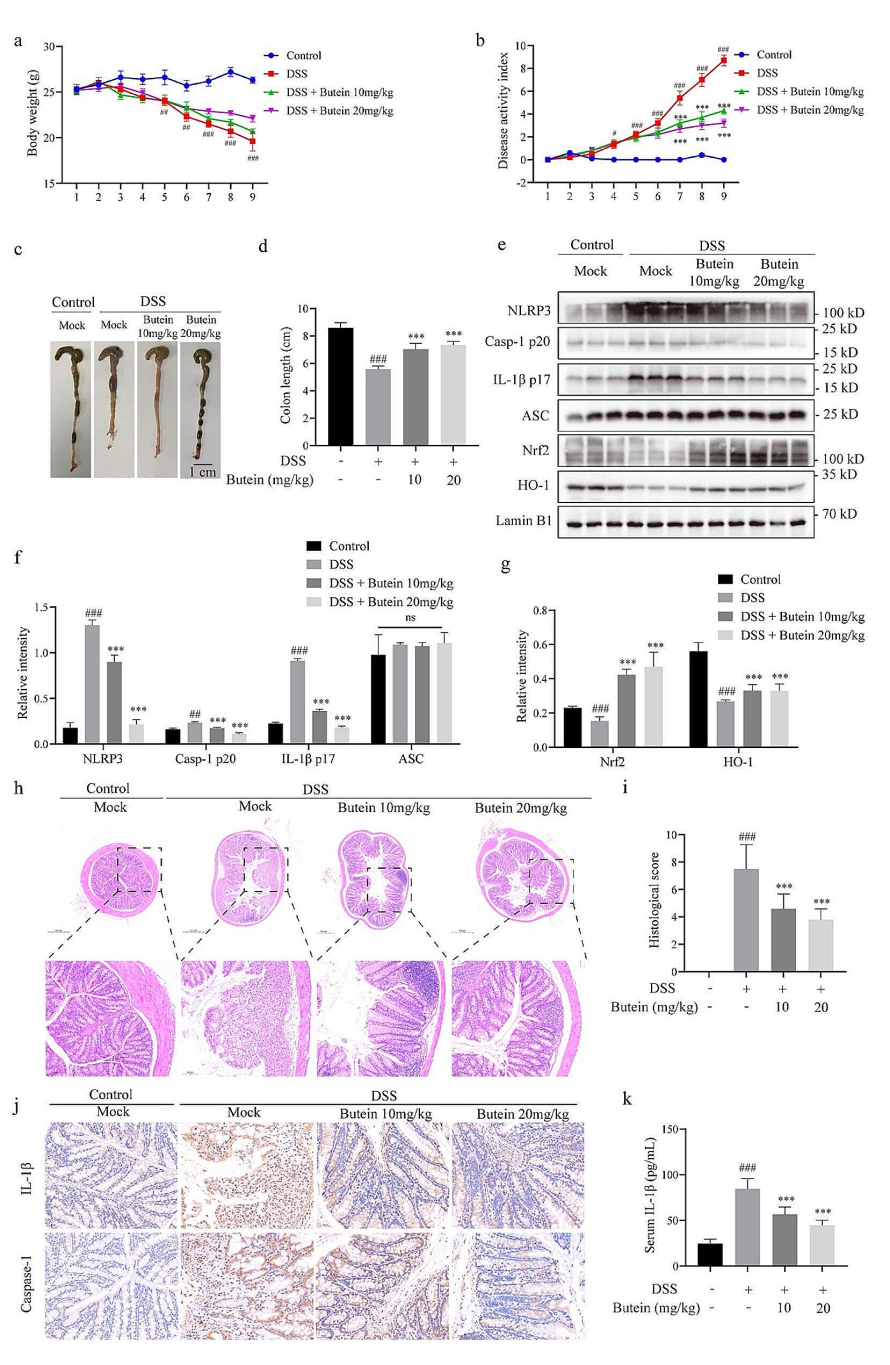
Butein alleviates DSS-induced colitis model. **a-d** DSS was dissolved in daily drinking water to induce colitis in C57BL/6 male mice for 9 days. Mice received butein (10, or 20 mg/kg) by intragastric gavage during the modeling period. Body weight change (**a**), DAI (**b**), representative colon image (**c**) and the colon lengths (**d**) of the mice were evaluated (*n* = 10 for each group). **e** Western blot analysis of caspase-1 p20, IL-1β p17, NLRP3, ASC, Nrf2, and HO-1 in colon tissues after homogenization of protein content. **f, g** Relative intensity of caspase-1 p20, IL-1β p17, NLRP3, ASC (**f**), and Nrf2 and HO-1(**g**). **h, i** HE staining (scale bar: 500/100 µm) (**h**) and histological score in colon sections (**i**). **j** Immunohistochemical staining (scale bar: 50 μm) of IL-1β and caspase-1 in colon sections. **k** ELISA analysis of serum IL-1β production. ^#^*P* < 0.05, ^##^*P* < 0.05, and ^###^*P* < 0.001 vs. the group of Control. ^*^P < 0.05, ^**^P < 0.01, and ^***^P < 0.001 vs. the group of DSS

Next, we investigated whether butein could inhibit NLRP3 inflammasome activation in vivo. Previous studies have shown that DSS-induced colitis in mice is an inflammatory disease associated with NLRP3 inflammasome [33]. We allowed mice to drink 2.5% (wt/vol) DSS ad libitum to verify whether butein could alleviate DSS-induced colitis and NLRP3 activation in vivo. The results showed that DSS induced severe colitis in mice characterized by diarrhea, intestinal bleeding, weight loss and shortened colon length (Fig. 5a-d). Compared with those in the DSS group, butein-treated mice exhibited less weight loss and a lower DAI score, a key parameter indicative of colitis severity (Fig. 5b). Moreover, butein reversed DSS-induced colon shortening (Fig. 5c, d). Furthermore, detailed histological evaluation of mouse colons exposed to DSS revealed severe pathological changes, including extensive disruption of crypt architecture, marked loss of goblet and epithelial cells, neutrophil and monocyte infiltration, and mucosal damage and necrosis, while butein markedly reversed the severity of pathological changes in colitis (Fig. 5h, i).

We then confirmed whether butein affects the activation of NLRP3 inflammasome in a colitis model. The results showed that the addition of DSS to the drinking water increased the levels of NLRP3, caspase-1 p20 and IL-1β p17 in mouse colon tissue, while butein prevented these cleavages (Fig. 5e, f), suggesting that butein blocks the activation of NLRP3 inflammasome in DSS-induced colitis. Immunohistochemical staining also revealed that butein inhibited the expression of caspase-1 and IL-1β in mouse colon tissue (Fig. 5j). In addition, ELISA demonstrated that butein notably inhibited the production of IL-1β in mouse serum (Fig. 5k). More importantly, mice lacking Nrf2 are more susceptible to DSS-induced colitis [34]. Our experiments showed that DSS induced the downregulation of Nrf2 and HO-1 levels in mouse colon tissue, while butein prominently activated antioxidant pathways to alleviate inflammatory responses (Fig. 5e, g). Taken together, these findings indicate that butein attenuates NLRP3 inflammasome activation, upregulates the Nrf2 pathway, and reverses the pathological process in a DSS-induced colitis model.

### Butein is effective in the HFD-induced NASH model

**Fig. 6:**
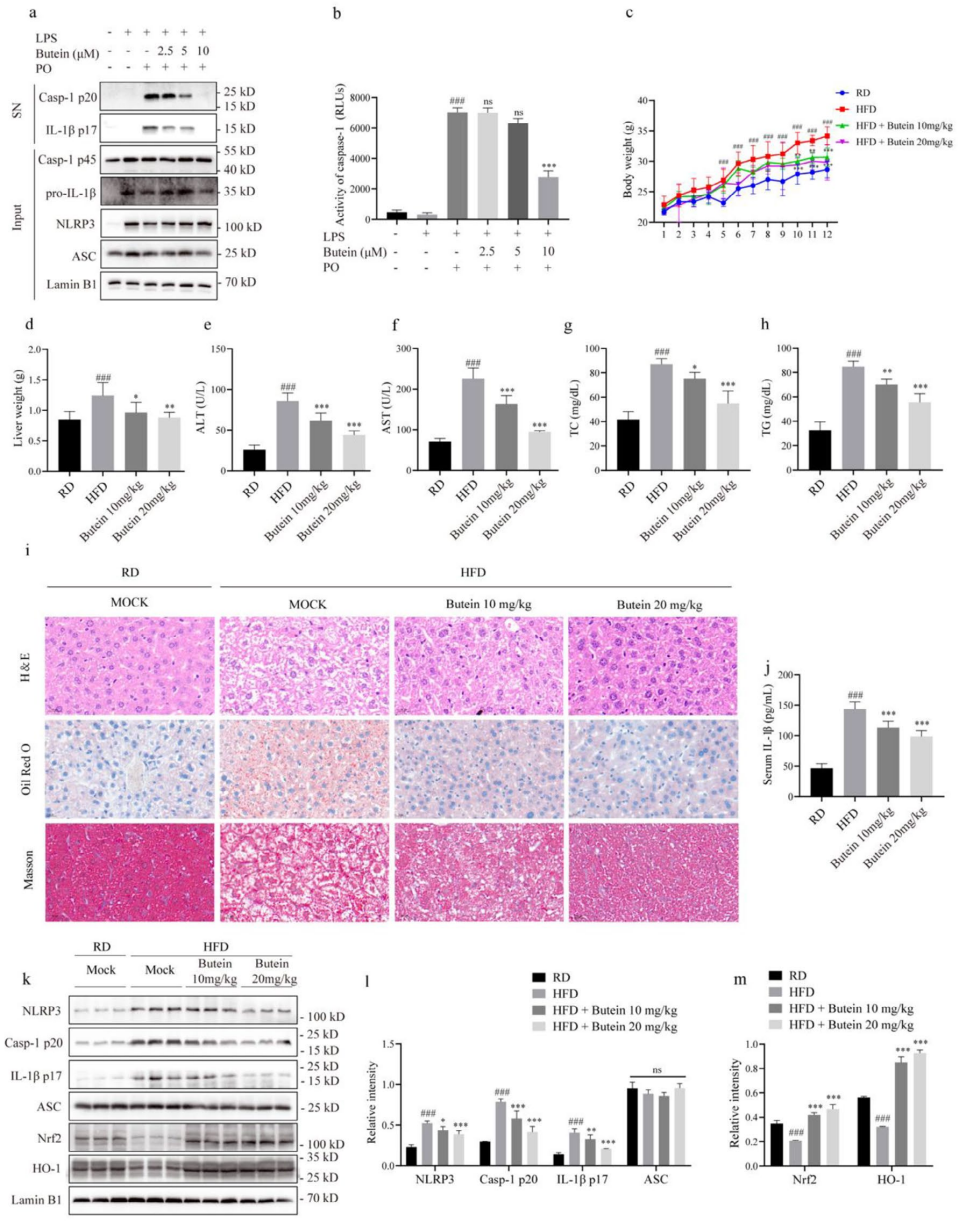
Butein is effective in HFD-induced NASH model. **a** LPS-primed BMDMs were treated with butein and then stimulated with PO (300 µM) for 6 h. Western blot analyses of pro-IL-1β, caspase-1 p45, NLRP3, and ASC in input and IL-1β p17, caspase-1 p20 in SN. **b** Caspase-1 activity in the SN. **c-h** HFD was used to induce a NASH model in C57BL/6 male mice for 12 weeks. At 9–12 weeks, mice received butein (10, or 20 mg/kg) by intragastric gavage. Changes in body weight (**c**), liver weight (**d**), ALT (**e**), AST (**f**), TC (**g**), and TG (**h**) were evaluated (*n* = 10 for RD and HFD groups, *n* = 5 for high and low dose of butein intervention groups). **i** HE staining, oil red O staining, and Masson’s staining (scale bar: 20 μm) of liver tissues. **j** ELISA analysis of serum IL-1β production. **k** Western blot analysis of caspase-1 p20, IL-1β p17, NLRP3, ASC, Nrf2, and HO-1 in colon tissues after homogenization of protein content. **l, m** Relative intensity of caspase-1 p20, IL-1β p17, NLRP3, ASC (**L**), and Nrf2 and HO-1 (**m**) as described in (**k**). ^###^*P* < 0.001 vs. the group of RD. ^*^P < 0.05, ^**^P < 0.01, and ^***^P < 0.001 vs. the group of HFD

Aberrant activation of NLRP3 inflammasome is thought to be an important mechanism driving HFD-induced NASH, and blocking NLRP3 inflammasome activation can reduce liver inflammation and fibrosis and ameliorate pathological changes in NASH [35]. A mixture of sodium palmitate and sodium oleate (PO) is commonly used to establish cellular models of NASH [36]. To test the efficacy of butein on HFD-induced NASH, we primed BMDMs with LPS and stimulated them with PO for 6 h after butein intervention. Cell lysates and supernatants were analyzed for the presence of cleaved and full-length caspase-1 and IL-1β by immunoblotting. After stimulation with PO, butein blocked the cleavage of caspase-1 and IL-1β (Fig. 6a and Supplementary Fig. 1c). Caspase-1 activity in cell supernatants was enhanced by PO but was effectively inhibited by high concentrations of butein (Fig. 6b). In conclusion, butein effectively blocks LPS-primed and PO-stimulated NLRP3 inflammasome activation.

A HFD was then used to construct a NASH mouse model and test whether butein could alleviate liver lesions. Compared with the RD, the HFD not only rapidly increased the body weight of the mice but also increased the size and weight of the liver. Surprisingly, the increases in body weight and liver weight were suppressed by butein administration (Fig. 6c, d). Moreover, compared with RD-fed mice, HFD-fed mice exhibited significant hepatic morphological changes, such as hepatic steatosis, inflammation, fibrosis, and elevated plasma ALT, AST, TC, and TG levels. As expected, butein treatment provided significant relief from the above pathological changes (Fig. 6e-i). Next, we investigated whether the ability of butein to ameliorate NASH lesions was correlated with its ability to block NLRP3 inflammasome activation. The results showed that butein inhibited the levels of NLRP3, caspase-1 p20 and IL-1β p17 in the livers of NASH mice (Fig. 6k, l). The ELISA results also showed that butein blocked HFD-induced serum IL-1β production (Fig. 6j). Hepatocyte-specific Nrf2 activation has been shown to prevent NASH by inhibiting lipogenesis, supporting mitochondrial function, modulating inflammation, and adapting to HFD diet-induced oxidative stress [37, 38]. Therefore, we investigated whether butein has potential beneficial effects on HFD-induced NASH by modulating the antioxidant pathway Nrf2. HFD consumption downregulated the protein levels of Nrf2 and its downstream HO-1 in liver tissue, while butein notably activated the antioxidant pathway to alleviate the inflammatory response (Fig. 6k, m). Collectively, these results suggest that butein attenuates hepatic lipid accumulation, inflammation, and fibrosis by inhibiting NLRP3 inflammasome activation, thereby effectively preventing the progression of HFD-induced NASH.

### Butein ameliorates LPS-induced peritonitis

**Fig. 7:**
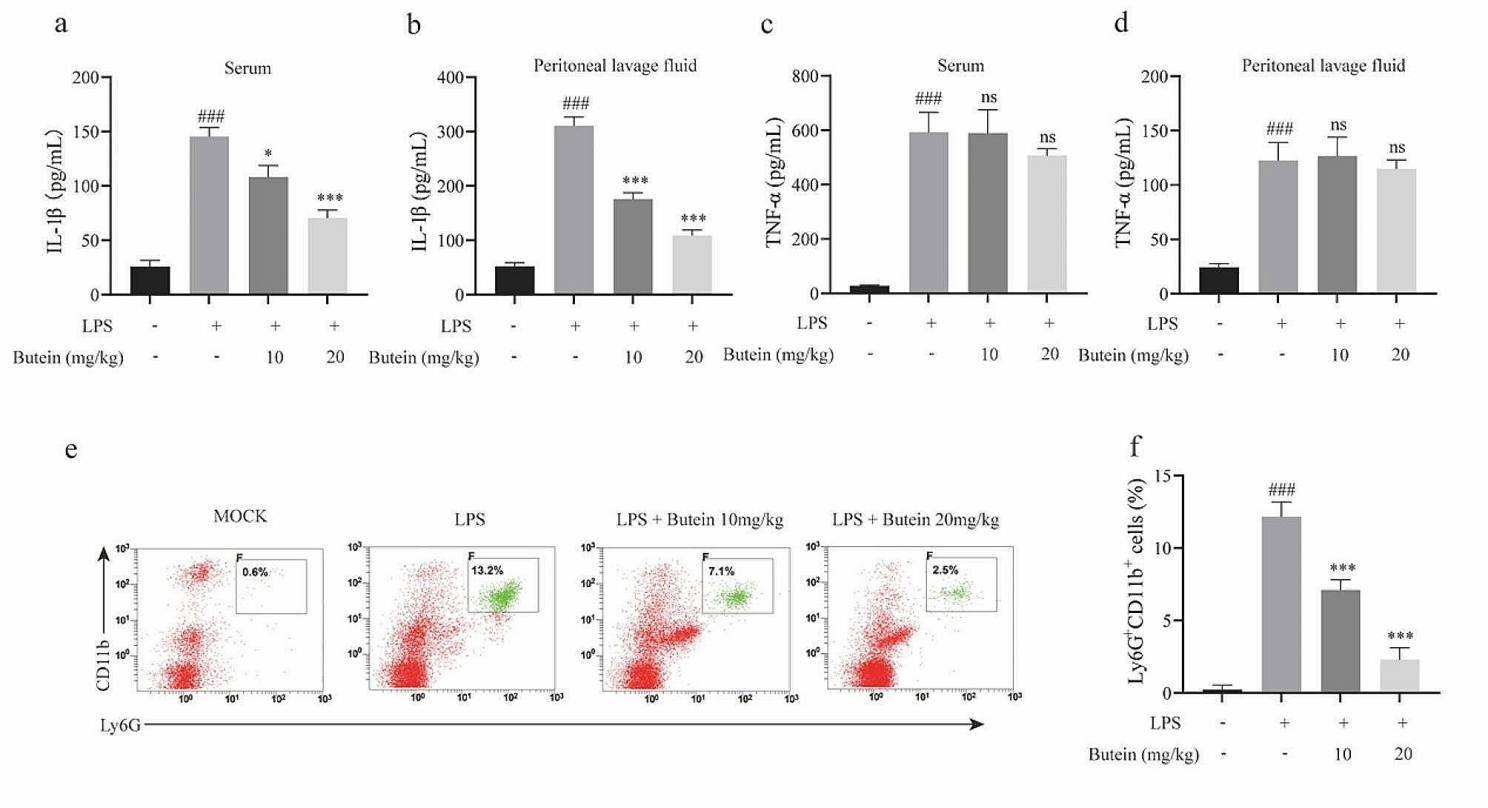
Butein ameliorates LPS-induced peritonitis. **a-d** ELISA analysis of IL-1β production in serum (**a**) and peritoneal lavage fluids (**b**), and TNF-α production in serum (**c**) and peritoneal lavage fluids (**d**). **e, f** Flow cytometric analysis of the proportion of neutrophils in peritoneal lavage fluid (Ly6G^+^CD11b^+^). ^###^*P* < 0.001 vs. the group of control. ^*^P < 0.05 and ^***^P < 0.001 vs. the group of LPS

Peritonitis induced by intraperitoneal injection of LPS is NLRP3-dependent and characterized by IL-1β production and neutrophil influx [39, 40]. We further evaluated the efficacy of butein in a mouse model of LPS-induced peritonitis. Mice were pretreated with butein for 1 h and subsequently injected intraperitoneally with LPS. Two hours later, the production of proinflammatory cytokines in the peritoneal lavage fluid and serum was measured. The results showed that IL-1β production after LPS injection was increased in the serum and peritoneal lavage fluid of mice than in those of control mice, whereas butein effectively decreased the production of IL-1β in LPS-induced peritonitis model mice, but had no significant effect on the production of TNF-α, a cytokine that is independent of inflammasome (Fig. 7a-d). In addition, consistent with the inhibitory effects on proinflammatory cytokines secretion, butein pretreatment reduced the proportion and number of neutrophils in the peritoneal lavage fluid of the mice (Fig. 7e, f). These data suggest that butein ameliorates LPS-induced peritonitis in vivo.

## Discussion

Research has shown that different diseases may have similar pathogenic mechanisms during development; thus, the same method or drug can be used to treat different diseases (known as “homotherapy for heteropathy”) with excellent efficacy, and the key to this is the discovery of mechanisms that are common to different diseases [1, 2, 41]. Although NLRP3 inflammasome is an important sentinel of the host’s innate immune defense [42–44], evidence suggests that its aberrant activation is involved in the onset and progression of a wide range of inflammatory and autoimmune diseases, including NASH [5], colitis [45, 46], and peritonitis [47, 48], which are the subjects of this study. Therefore, given the role of NLRP3 inflammasome in the pathogenesis, blocking its activation is considered a potential common strategy for treating these diseases. In the present study, we demonstrated that the natural product butein inhibits NLRP3 inflammasome activation both in vitro and in vivo and may be a valid candidate for the treatment of NLRP3-mediated inflammatory diseases. In addition, activation of NLRC4 and AIM2 inflammasomes also leads to caspase-1 activation and IL-1β secretion [49]. Our study revealed that butein similarly inhibited the activation of AIM2 and NLRC4. AIM2 inflammasome has been reported to play a role in inflammatory diseases, cancer, and infections [50–52]. Aberrant activation of NLRC4 is linked to infectious diseases, autoinflammation, and cancer in humans [8, 53, 54]. Taken together, these findings indicate that butein is a broad-spectrum inhibitor of inflammasomes and may be superior for the treatment of NLRP3-, AIM2- and NLRC4-driven diseases.

Numerous studies have shown that there is an interaction between inflammasome and the Nrf2 signaling pathway at several levels [55]. ROS may in part be the link between the two pathways. ROS act as agonists to activate NLRP3 inflammasome [56, 57], whereas Nrf2 responds to endogenous and exogenous stress induced by ROS or electrophiles by inducing antioxidant Nrf2 target genes, such as HO-1 and NAD(P)H: quinone oxidoreductase 1, to eliminate oxidative stress and inflammation [57, 58]. Recent studies have shown that natural phytochemicals such as magnolol, gallic acid, and rhein can inhibit IL-1β secretion and oxidative damage by upregulating Nrf2-mediated expression of NAD(P)H: quinone oxidoreductase 1 and HO-1, suggesting that Nrf2 may be a common potential target for therapeutic modulation of NLRP3-related disorders [59–61]. In the present study, butein inhibited ROS-induced NLRP3 inflammasome activation by upregulating the expression of Nrf2 and its downstream effector HO-1. Moreover, butein attenuated NLRP3 inflammasome-driven disease progression by upregulating Nrf2/HO-1 in DSS-induced colitis and HFD-induced NASH models.

Non-alcoholic fatty liver disease (NAFLD) is a clinicopathological syndrome caused by factors other than alcohol and other well-defined liver-damaging factors and is characterized by excessive fat deposition, inflammation, and an imbalance of redox homeostasis in hepatocytes. NAFLD is an abnormal liver process ranging from hepatic steatosis to NASH that can lead to cirrhosis, liver failure, and even hepatocellular carcinoma [62]. In the present study, we found that PO, a mixture of sodium palmitate and sodium oleate, stimulated the expression of high levels of caspase-1 p20 and IL-1β p17 in LPS-primed BMDMs, whereas butein effectively blocked the cleavage of caspase-1 and IL-1β. Furthermore, butein markedly attenuated HFD-induced hepatic steatosis, inflammation, fibrosis, and other pathological changes in the HFD-induced NASH model. Most importantly, butein effectively blocked the expression of caspase-1 p20 and IL-1β p17 in liver tissue, suggesting its inhibitory effect on NLRP3 inflammasome. Targeting NLRP3 inflammasome is a promising therapeutic strategy that warrants further preclinical and clinical investigation. Amidst the euphoria over the progress of the NLRP3 inhibitor butein in NASH, parallel efforts must be made to assess safety during the course of therapy.

In conclusion, this study showed that butein is a potent NLRP3 inflammasome inhibitor with significant therapeutic effects on NLRP3-driven inflammatory diseases; i.e., peritonitis, colitis, and NASH. We hope that our study will illustrate the connectivity of heterogeneous diseases and facilitate the development of new drugs by using one drug to treat multiple diseases via the same mechanism.

### Electronic supplementary material

Below is the link to the electronic supplementary material.

Supplementary Material 1

Supplementary Material 2

## Data Availability

No datasets were generated or analysed during the current study.
