# Supplementary figures and images for: Homotherapy for heteropathy: therapeutic effect of Butein in NLRP3-driven diseases
Source: Cell Commun Signal. 2024 Jun 7;22:315. doi: 10.1186/s12964-024-01695-7 (PMC11158000; doi:10.1186/s12964-024-01695-7)

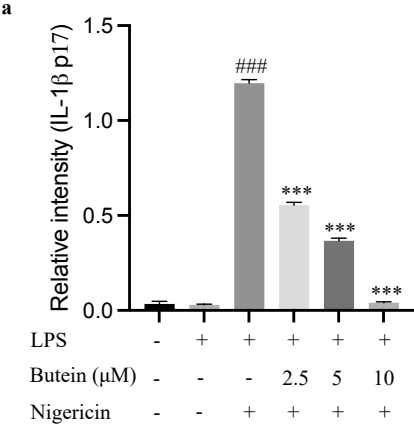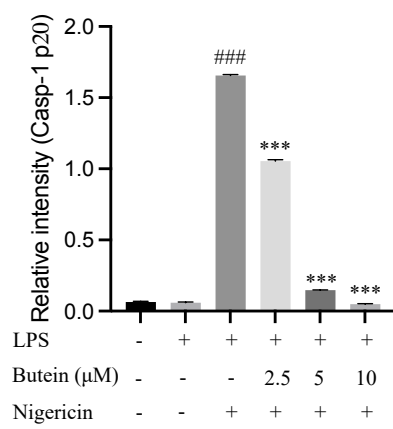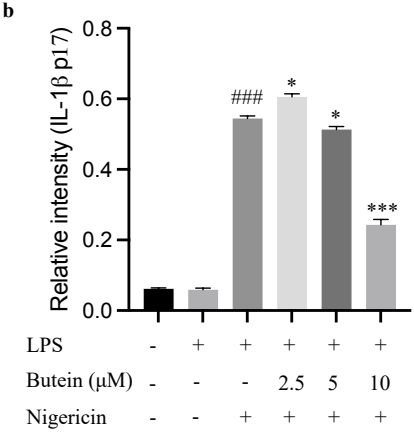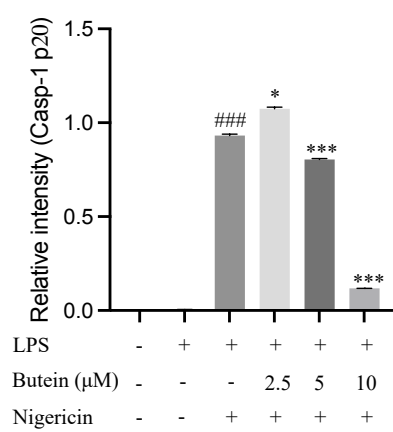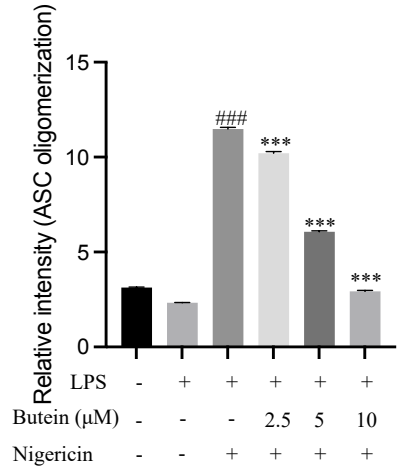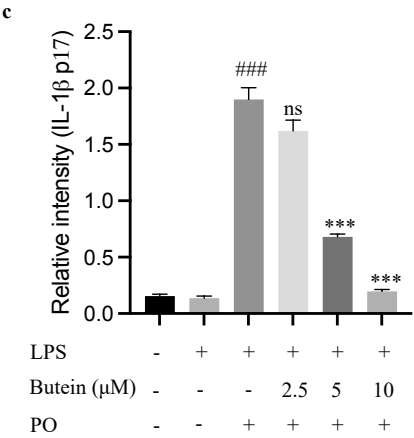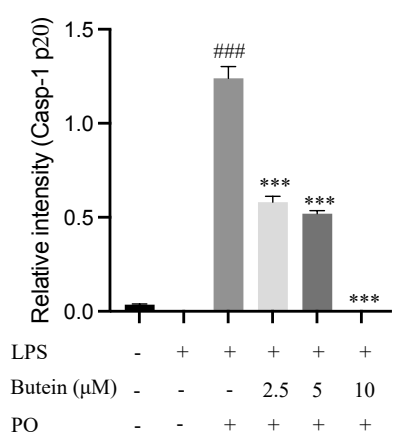

Supplement: Supplementary file 1 — Supplementary Material 1 [file 12964_2024_1695_MOESM1_ESM.pdf]
