# Supplementary material for: Homotherapy for heteropathy: therapeutic effect of Butein in NLRP3-driven diseases
Source: Cell Commun Signal. 2024 Jun 7;22:315. doi: 10.1186/s12964-024-01695-7 (PMC11158000; doi:10.1186/s12964-024-01695-7)
Supplement: Supplementary file 2 — Supplementary Material 2 [file 12964_2024_1695_MOESM2_ESM.docx]

**Supplementary Fig. 1. Relative intensity of western blot analysis. a** BMDMs were primed with LPS for 4 h and then treated with butein for 1 h prior to stimulation with nigericin for 1 h. Western blot analyses of IL-1β p17 and caspase-1 p20 in SN. Relative intensity of IL-1β p17 and caspase-1 p20. **b** LPS-primed BMDMs were treated with butein for 1 h and then stimulated with nigericin for 1 h. Western blot analysis of cross-linked ASC in the Triton X-insoluble pellet. Relative intensity of IL-1β p17, caspase-1 p20, and ASC oligomerization. **c** LPS-primed BMDMs were treated with butein and then stimulated with PO (300 μM) for 6 h. Western blot analyses of IL-1β p17 and caspase-1 p20 in SN. Relative intensity of IL-1β p17 and caspase-1 p20. ^###^P < 0.001 vs. the group of LPS. ^*^P < 0.05, and ^***^P < 0.001 vs. the group of LPS + nigericin/PO.
